# Supplementary material for: Anisotropic magnon damping by zero-temperature quantum fluctuations in ferromagnetic CrGeTe3
Source: Nat Commun. 2022 Jul 12;13:4037. doi: 10.1038/s41467-022-31612-w (PMC9276656; doi:10.1038/s41467-022-31612-w)
Supplement: Supplementary file 1 — Supplementary Information [file 41467_2022_31612_MOESM1_ESM.pdf]

Supplemental Information for Anisotropic magnon damping by zero-temperature quantum fluctuations in ferromagnetic $CrGeTe_3$

1 Elastic neutron scattering to check sample quality

1.1 Sample mosaicity

Figures S1(a) - S1(d) show the elastic parts of the time-of-flight neutron scattering data measured using the SEQUOIA and the AMATERAS spectrometers, respectively, which are cut over the selected high-symmetry planes of the reciprocal space. The integration range in momentum space for different cuts in main text and supplementary information is summarized in Table S1. The mosaic spreads of the co-aligned crystal samples for these measurements are shown in Figs. S1(f) - S1(i) as the transverse momentum-dependent intensity distributions of the Bragg peaks on the neutron scattering experiments using the SEQUOIA and AMATERAS experiment mentioned in the main text. The mosaic spread of the sample measured at the SEQUOIA spectrometer was 8.9° in full-width-half-maximum (FWHM) in the plane and 6.5° out of the plane. The sample measured at the AMATERAS spectrometer had the mosaic spread of 10.5° in FWHM in the plane and 4.1° out of the plane.

1.2 Ferromagnetic order parameter

Figure S1(e) shows the ferromagnetic order parameter measured in the form of the $(1, 1, 0)$ Bragg peak intensity, which was collected using the HB-3 triple-axis spectrometer at the High Flux Isotope Reactor, Oak Ridge National Laboratory. Its temperature dependence was fitted by a power law function

$$I = I_m \left(\frac{T_C - T}{T_C} \right)^{2\beta} + I_n,$$

where as I_m and I_n are the magnetic and nuclear scattering intensities, respectively. The best-fit parameters were obtained to be $T_C = 65$ K and $2\beta = 0.41$.

1.3 Broadening effect from non-SLC origins

The apparent broadening of the in-plane spin excitations may appear due to multiple origins that are not related to the SLC. Some of the possible origins include (a) instrumental resolution, (b) integration over dispersive excitations along out-of-plane $[0\ 0\ L]$, and (c) mosaic spread of the sample. In Table S1, we list all the integration ranges of the experimental data plots, if not specified in the text. We have estimated the non-SLC broadening using the Monte-Carlo method while taking all three contributions above into account. We used the following recipe to calculate broadening observed, for example, along the $[H,H]$ direction in the data integrated between $0 \leq L \leq 5$. First, we prepared two wave vectors, $[0,0,L]$ and $[1,1,L]$, with a randomly selected L index within the desired range (see red arrows in the left panel of Fig. S2(a)). After being converted to the instrumental (xyz) coordinate, they were multiplied by the 3D rotational matrix $R = R_z R_y R_x$, in which

$$R_x = \begin{bmatrix} 1 & 0 & 0 \\ 0 & \cos\theta_x & -\sin\theta_x \\ 0 & \sin\theta_x & \cos\theta_x \end{bmatrix}, R_y = \begin{bmatrix} \cos\theta_y & 0 & \sin\theta_y \\ 0 & 1 & 0 \\ -\sin\theta_y & 0 & \cos\theta_y \end{bmatrix}, , R_z = \begin{bmatrix} \cos\theta_z & -\sin\theta_z & 0 \\ \sin\theta_z & \cos\theta_z & 0 \\ 0 & 0 & 1 \end{bmatrix}.$$

Above, θ_x, θ_y, and θ_z are the rotation angles, which were selected based on the Gaussian probability of $p(\theta_x, \theta_y, \theta_z) = \frac{(2\pi)^{-3/2}}{\sigma_x \sigma_y \sigma_z} e^{-\theta_x^2/2\sigma_x^2} e^{-\theta_y^2/2\sigma_y^2} e^{-\theta_z^2/2\sigma_z^2}$. For the SEQUOIA data, $\sigma_x = \sigma_y = 2.8°$, and $\sigma_z = 3.8°$ were used, which were estimated from the out-of-plane and in-plane FWHM, respectively, of the sample mosaic spread. After being converted back to the sample's (H, K, L) coordinate, magnon energies and intensities were calculated over an array between the two wave vectors (see red arrows and a green line between them in the right panel of Fig. S2(a)). Finally, the convolutions with instrumental energy resolution was performed at each wave vector. The above process was repeated $N \geq 1000$ times and integrated along L index to quantitatively estimate the broadening effect.

It is important to note that the interlayer couplings may render a considerable uncertainty to the amount of apparent excitation broadening. In this work, we assumed that there is only one type of $J_c = -0.86$ meV($= J_{c1}$ in Fig. S2(b)) in $CrGeTe_3$. As shown in Fig. S2(b), however, there is another interlayer exchange, J_{c2}, in honeycomb ferromagnets with $R\bar{3}$ symmetry, which is known to be important in CrI_3. While J_{c1} and J_{c2} compete to determine L-dependent dispersions, the uncertainty remains as only the low-energy mode was clearly observed due to the excessive broadening of the high energy mode. We thus compare two possible cases resulting in the same out-of-plane bandwidth of $W = 2.58$ meV: 1) $J_{c1} = 0$ and $J_{c2} = -W/18S = -0.096$ meV; 2) $J_{c1} = -W/2S = -0.86$ meV and $J_{c2} = 0$. In both cases, we assume that neither exchange is antiferromagnetic as there is no evidence for such. The plots in Figs. S2(c) and S2(d) compare the results of the Monte Carlo calculations in the two cases including the sample mosaic spread and the instrumental resolution. We find that the excitation broadening is smaller at all wave vectors with 1) $J_{c1} = 0$ than 2) $J_{c2} = 0$. For instance, the case 1) has the equal bandwidth of 2.58 meV for both magnon modes along the [0,0,L], [1/2,1/2,L], and [1,0,L] directions, respectively. In comparison, the case 2) has the smaller bandwidth of 0.86 meV along the [1/2,1/2,L], and [1,0,L] directions. We thus conclude that our model with J_{c1} only will not underestimate the broadening due the L-dependent dispersions.

Figures S2(e) and S2(f) show the constant-Q cuts at $(H,K) = (1/2,1/2)$ and (1,0), respectively, of the Monte Carlo calculations overplotted with the inelastic neutron scattering intensities. It is clearly shown that the experimental intensities are significantly broader than the calculations at both wave vectors. At $(1, 0)$, in particular, the broad intensity extended down to ≈ 20 meV is far from the calculations. These results demonstrate that the magnon broadening observed in $CrGeTe_3$ is not sole attributed to trivial non-SLC origins.

The sample mosaic effect is also considered in the phonon simulation with Monte Carlo method as shown in Fig. 2(e,f) of the main text. The sample mosaic is measured with AMATERAS of 10.5 degrees in-plane and 4.1 degrees out-of-plane. In this simulation, a Gaussian distribution function is used to describe the sample mosaic in $\mathbf{Q}$ space, and 1000 random $S(\mathbf{Q}, E)$ slices that follow the distribution centered at the $\mathbf{Q}$ path of interest are generated and averaged to obtain the final $S(\mathbf{Q}, E)$.

2 Additional inelastic neutron scattering results

Figure S3(a) shows the spin waves along the in-plane $[H, H, 4.5]$ direction. Note that the excitations along the $[H, H, 3]$ direction, which covers the zone-center Γ point at $Q = (0, 0, 3)$, was already shown in Fig. 1(f) of the main text. Figure S3(b) shows the spin anisotropy gap of $\Delta = 0.098$ meV at the Γ point, from which the easy-axis anisotropy field $D_z = \Delta/2S = 0.033$ meV can directly be obtained for the ferromagnet.

Figure S3(c) shows the low-energy inelastic neutron scattering intensities along the out-of-plane $[0, 0, L]$ direction. The excitations observed at the low Q range below $L \leq 9$ are the spin waves, which were

also shown in Fig. 1(e) of the main text. In contrast, the excitations observed above $L \geq 12$ are the longitudinal acoustic phonons, which were also shown in Fig. 2(e) of the main text.

Figure S3(d,e) show the unfolded color plot of the $[H,H]$ dispersion at 3.5 K and 55 K, respectively. The linewidth has less than 5% difference between the left and right branch of the spin excitation, therefore the broadening of the spin excitation is not an experimental artifact caused by resolution ellipses. For the 55 K dispersion, we use J_1 = -2.57meV, J_2 = -0.15meV, J_3 = -0.19meV, J_c = -0.70meV, $\mathbf{A}$ = 0.25meV and D_z = -0.03meV for the LSWT simulation in Fig. 4 of the main text.

Figure S4 shows the raw data of the constant-Q cuts in Fig. 3(c) of the main text, and another set of constant-Q cuts along the $[0, 0, L]$ direction for comparison. Gaussian fits (red lines) are plotted on top of the raw data (black dots). The gap at $L = 3.7$ in Fig. S4(d) is due to a detector gap which is also shown in Fig. 1(e) of the main text. All plots are normalized according to the Gaussian fits.

3 Local dynamic spin susceptibilities, magnetization, lattice parameters, and refinements

Figures S5(a) and S5(b) show the energy-dependent local dynamic spin susceptibility, $\chi''(E)$, at $T = 7$ and 50 K, respectively, integrated over the finite wave vector ranges marked inside the plots. In Fig. S5(a), the integration was done around $Q = (0, 0, 3)$ at the zone center over the wide range along the $[H, H, 3]$ direction to cover the in-plane spin waves. In Fig. S5(b), the integration was done between $Q = (0, 0, 4.5)$ and $(0, 0, 6)$ to cover the out-of-plane spin waves. The in-plane $\chi''(E)$ was reduced significantly at higher temperature (T = 50 K) over the entire energy range observed (see Fig. S5(a)). In contrast, the out-of-plane $\chi''(E)$ exhibited a noticeable change only near the band top ($E \geq$ 2.2 meV), which is ascribed to the renormalization of the spin wave band width (see Fig. S5(b)).

Figure S5(c) shows the temperature dependence of the in-plane (a) and out-of-plane (c) lattice parameters, respectively, which were observed using the BL-9 CORELLI diffractometer at the Spallation Neutron Source, Oak Ridge National Laboratory. It reveals the opposite temperature dependence between a and c as well as a strong spin-lattice coupling across T_C = 65 K.

Figure S5(d) show the magnetization data on $CrGeTe_3$. A Curie-Weiss law fit is done on the $M - T$ graph considering data between 200 and 300 K, yielding a T_{CW} of 66.1 K. The negative deviation from the Curie-Weiss law below 150 K is consistent with the picture that the ferromagnetic exchange coupling strengthens with decreasing temperature. The saturation moment is $\sim 3.2\mu_B$ per Cr atom (inset of Fig. S5(d)), consistent with the previous reports.

Figure S6 shows the const-E cuts of local dynamic susceptibility χ'' along in-plane and out-of-plane directions. Along the in-plane directions, the χ'' is reduced at 50 K compared with 7 K while it keeps the same along the out-of-plane direction.

Figure S7 shows the refinement results for data measured on the TOPAZ single crystal diffractometer at the Spallation Neutron Source, Oak Ridge National Laboratory, with over 3000 Bragg peaks at 5 K, 70 K and 150 K. The refinement at 5 K yields $3.16\mu_B$/Cr magnetic moment, consistent with the $S = 3/2$ picture. The detailed refinement result is shown in Table 1 of the main text. The refinement result shows $<$ 2% Cr deficiency and $<$ 3% Te deficiency, proving the excellent sample quality. The disorder effect on the crystal caused by atom deficiency should have an isotropic effect on spin wave broadening, which we do not find in the resolution limited [0, 0,L] dispersion. In Figure 1(d) of the main text, we show the temperature dependence of the atomic displacement calculated by DFT and refinement results. The results qualitatively agree with each other, and both results give around 15% $\sim$ 25% J fluctuations at 5 K and $\sim$ 35% J fluctuation at 55 K, consistent with the simulated broadening effect in the dispersion.

4 Total moment sum rule

The sum rule of the dynamical structure factor $S(\mathbf{Q},\omega)$ for paramagnetic scattering is given as follows:

$$\frac{\int_{-\infty}^{\infty}\int_{BZ}\sum_{\alpha}S^{\alpha\alpha}(\mathbf{Q},\omega)d\mathbf{Q}dE}{\int_{BZ}d\mathbf{Q}} = g^2S(S+1)$$

where $\alpha = x, y, z$ and BZ stands for the Brillouin zone. The sum is integrated over the whole energy range and is averaged over the first Brillouin zone. The dynamical structure factor contains both the elastic and inelastic parts. At zero temperature where all spins are ordered, the elastic magnetic structure factor of the Bragg peaks integrates to S^2. Correspondingly, the inelastic structure factor $S_{xx}+S_{yy}+S_{zz}$ integrates to S. In neutron scattering experiments, however, neutrons interact only with the magnetic moments that are perpendicular to the momentum transfer $\mathbf{Q}$. Thus neutron scattering measures only the structure factors that are perpendicular to the spins:

$$S_{\perp} = \sum_{\alpha}(1-\bar{Q}_{\alpha}^2)S^{\alpha\alpha}$$

Above, $\bar{Q}_{\alpha}$ is the projection of the unit momentum transfer vector $\bar{\mathbf{Q}}$ along the $\alpha = x, y, z$ direction. The calculated $S_{\perp}$ should be proportional to the experimentally measured neutron scattering intensity $I(\mathbf{Q}, E)$:

$$S_{\perp}(\mathbf{Q},E) = \frac{13.77(\mathrm{barn}^{-1})}{g^2|f(\mathbf{Q})|^2e^{-2W}}I(\mathbf{Q},E),$$

where g is the Lande g-factor ($g \approx 2$), $f(\mathbf{Q})$ the magnetic form factor, and e^{-2W} the Debye-Waller factor which is sufficiently close to the unity at both 3.5 K and 55 K. We also note that the above equation was written with $I(\mathbf{Q}, E)$ in the unit of barn/eV/Sr/f.u. and $S_{\perp}(\mathbf{Q}, E)$ in 1/eV/Sr/f.u.

Due to detector coverage limits, not all the ($\mathbf{Q}$,E) points in the hexagonal FBZ (hexagon in [H K] plane cornered at the K point $(1/3, 1/3)$, $L = (-3/2, 3/2)$. Figure 1(c) of the main text shows the L-positive half.) can be reached. To map out a complete set of $I(E)$ up to 25meV, we averaged the experimental and theoretical intensities over the FBZ in the [H,K] plane and $L = (-6, 6)$. Figure S8 shows the difference between rhombohedral FBZ, hexagonal FBZ and our integration range. The magnetic form factor is corrected for the experimental data.

The experimental and theoretical INS intensity plot in Fig. 4(h) of the main text is $I(E) = I(\mathbf{Q}, E)/|f(\mathbf{Q})|^2$, integrated in the aforementioned $\mathbf{Q}$-space volume, and the theoretical value at 3.5K(55K) is multiplied by a factor that ensures its corresponding $S_{xx} + S_{yy} + S_{zz}$ equals 1.5(2.7) (See main text for details).

Figure	$[H\ H]$	$[-K\ K]$	L
1(e)	[-0.02, 0.02]	[-0.05,0.05]	N/A
1(f)	N/A	[-0.05,0.05]	[2.75,3.25]
2(e)	[-0.1, 0.1]	[-0.1, 0.1]	N/A
2(f)	[-0.1, 0.1]	N/A	[11.8,12.2]
4(a)	N/A	[-0.05,0.05]	[-5, 5]
4(b)	[-0.05,0.05]	N/A	[-6, 6]
4(c)	N/A	[-0.05,0.05]	[-5, 5]
4(d)	[-0.02, 0.02]	[-0.05,0.05]	N/A
4(e)	[0.45,0.55]	[-0.05,0.05]	[-5, 5]
4(f)	[-0.05,0.05]	[0.9,1.1]	[-6, 6]
S3(a)	N/A	[-0.05, 0.05]	[4.25, 4.75]
S3(c)	[-0.1, 0.1]	[-0.1, 0.1]	N/A
S3(d)	N/A	[-0.05,0.05]	[-5, 5]
S3(e)	N/A	[-0.05,0.05]	[-5, 5]
S4(a,b)	± 0.005	N/A	N/A
S4(c)	± 0.01	N/A	N/A
S4(d)	N/A	N/A	± 0.05
S6(a-d)	[-0.02, 0.02]	[-0.05, 0.05]	N/A
S6(e-j)	N/A	[-0.05, 0.05]	[2.8,3.2]

Table S1: **The integration range in the Q-space for the experimental plots.** Note the $[-K,K]$ direction is the same as the $[H,0]$ direction.

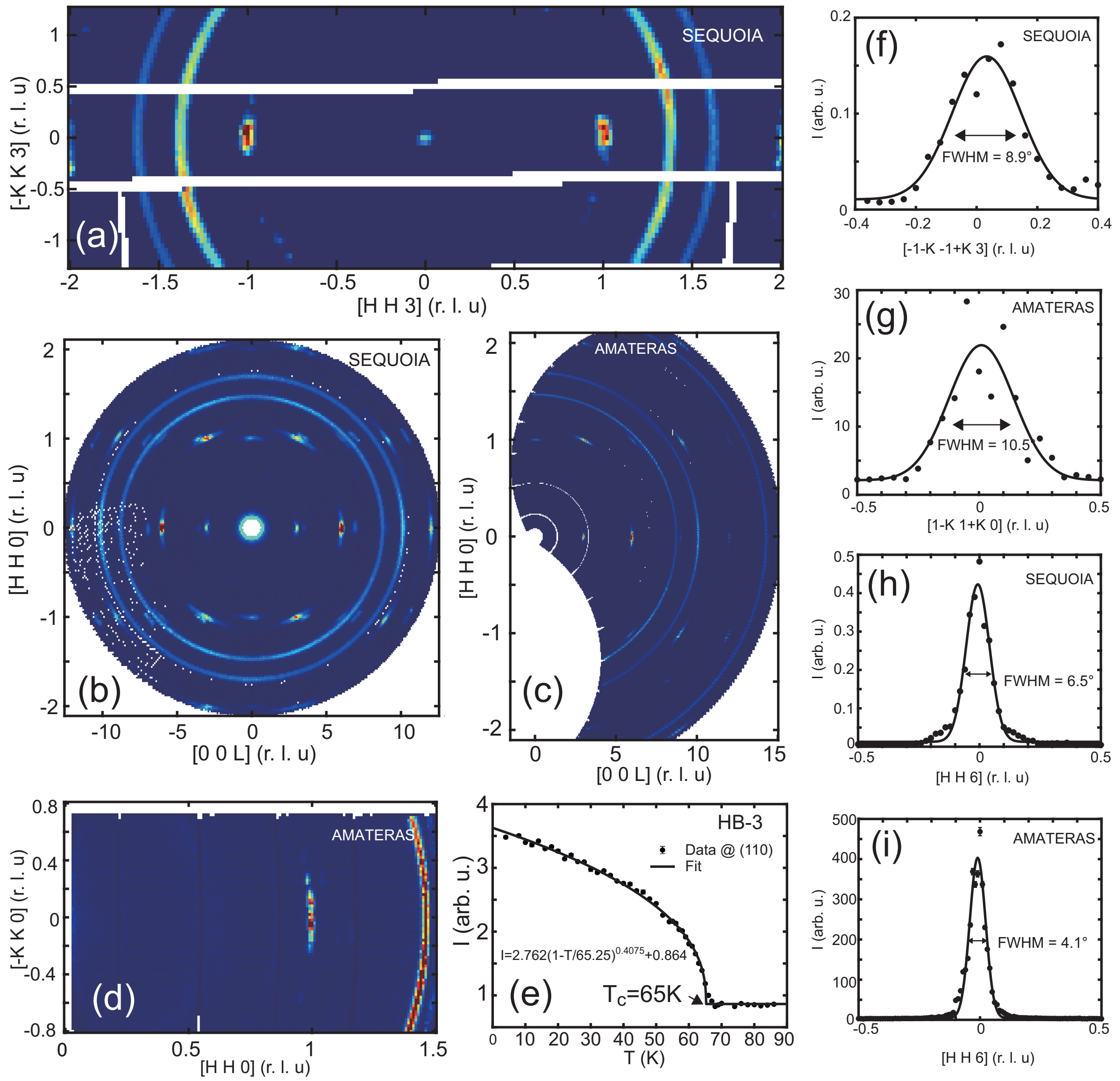

Figure S1: **The elastic parts from the time-of-flight neutron scattering data.** (a,b) 2D slices of the SEQUOIA data on the $[H, K, 3]$ and $[H, H, L]$ reciprocal planes, respectively. (c,d) 2D slices of the AMATERAS data on the $[H, H, L]$ and $[H, K, 0]$ reciprocal planes, respectively. (e) Temperature dependence of the $(1, 1, 0)$ Bragg peak intensity measured using the HB-3 spectrometer. The solid line is the powder law fit as discussed in the text. (f, g) The transverse intensity distributions of the $(1, 1, 3)$ Bragg peak in the SEQUOIA and AMATERAS data, respectively. The solid lines are Gaussian fits to estimate the in-plane mosaic spread. (h, i) The transverse intensity distributions of the $(0, 0, 6)$ Bragg peak in the SEQUOIA and AMATERAS data, respectively. The solid lines are Gaussian fits to estimate the out-of-plane mosaic spread.

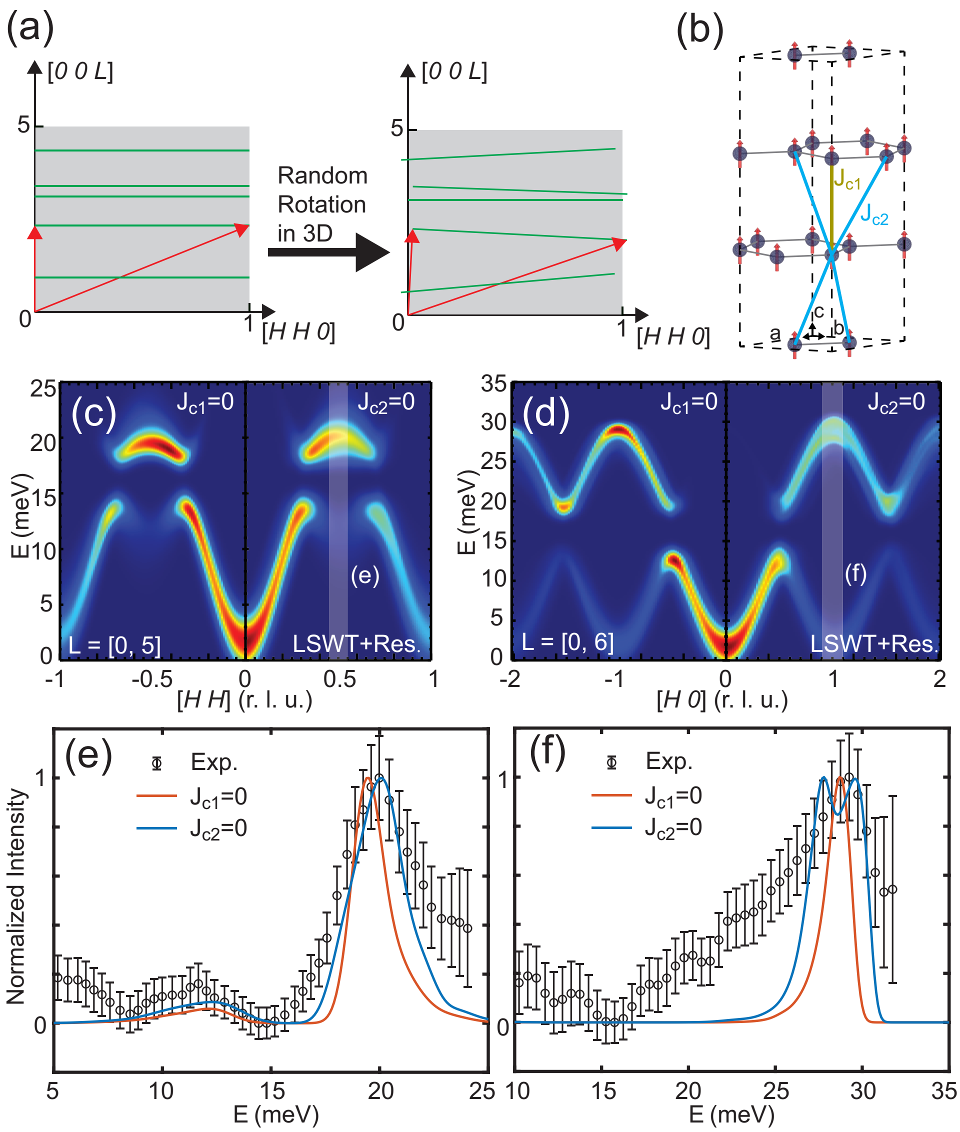

Figure S2: **Calculation on non-SLC broadening effects.** (a) Simplified schematics of the Monte-Carlo simulation along $[H\ H]$. (b) Illustration of interlayer exchange interactions J_{c1} (yellow) and J_{c2} (cyan). (c) Simulation results along $[H\ H]$ direction with L integrated between [0, 5], left panel shows the calculation result with (J_{c1} = 0, J_{c2} = -0.0955 meV), and right panel shows when (J_{c1} = -0.86 meV, J_{c2} = 0). (d) Same calculation along the $[H,0]$ direction with L integrated between [0, 6]. (e, f) Comparison of the SEQUOIA experimental result and the calculation in (c, d) at the M (e) and Γ (f) point, respectively. The Q-integration range is specified in the white shaded area in (c, d).

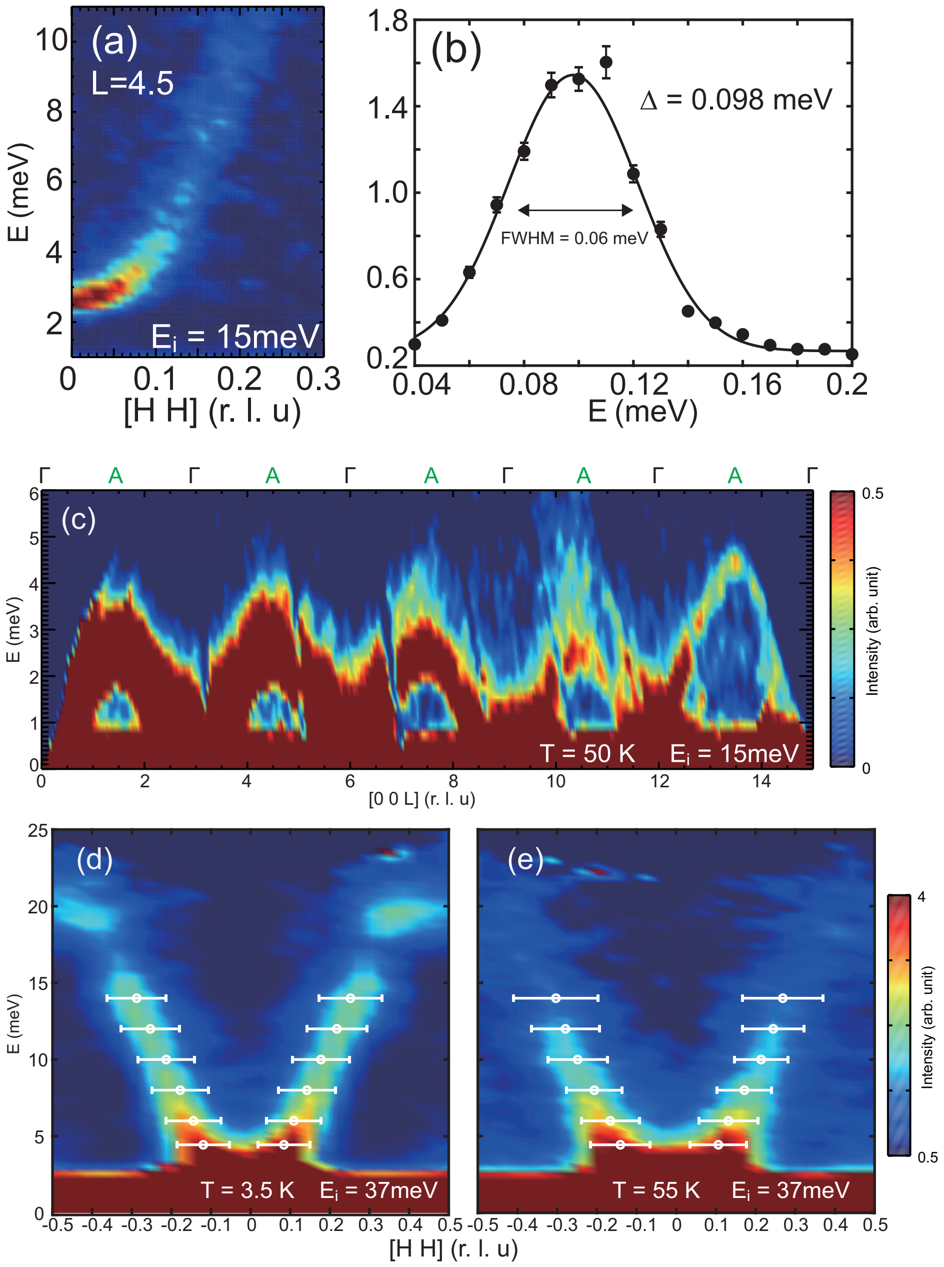

Figure S3: **Additional time-of-flight neutron scattering data at $T = 3.5$ K.** (a) The dispersion of the spin wave mode along the in-plane $[H, H]$ direction around the zone boundary at $Q = (0, 0, 4.5)$. Figure 3(c) of the main text includes the magnon linewidth of these data. (b) The energy dependence of the low-energy magnon mode at the zone center $Q = (0, 0, 3)$. The solid lines is the Gaussian fit, which gives the anisotropy gap energy at $\Delta = 0.098$ meV with the FWHM of 0.06 meV. (c) The inelastic excitations observed along the out-of-plane $[0, 0, L]$ direction simultaneously revealing magnons at $L \leq 9$ and L-LA phonons at $L \geq 12$. (d, e) Magnon dispersion along $[H\ H]$ at 3.5K and 55K, respectively. The white bars indicate the location and width (Gaussian-σ) of the const-E cuts.

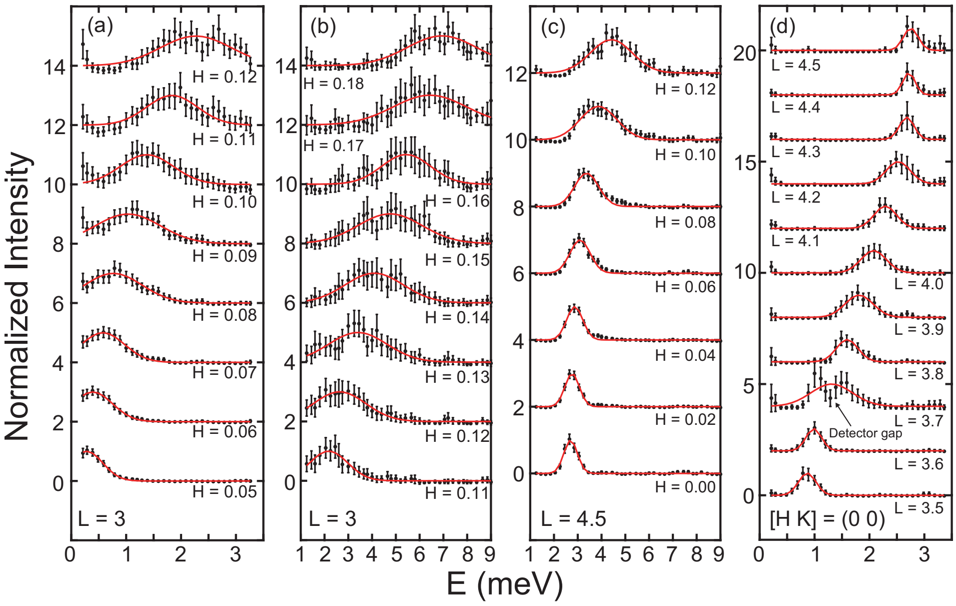

Figure S4: **The raw data of constant-Q cuts in Fig. 3(c) of the main text.** The integration range for (a,b) is $[-K, K] = (-0.05, 0.05)$, $L = (2.75, 3.25)$; for (c) is $[-K, K] = (-0.05, 0.05)$, $L = (4.25, 4.75)$; for (d) is $[H, H] = (-0.02, 0.02)$, $[-K, K] = (-0.05, 0.05)$.

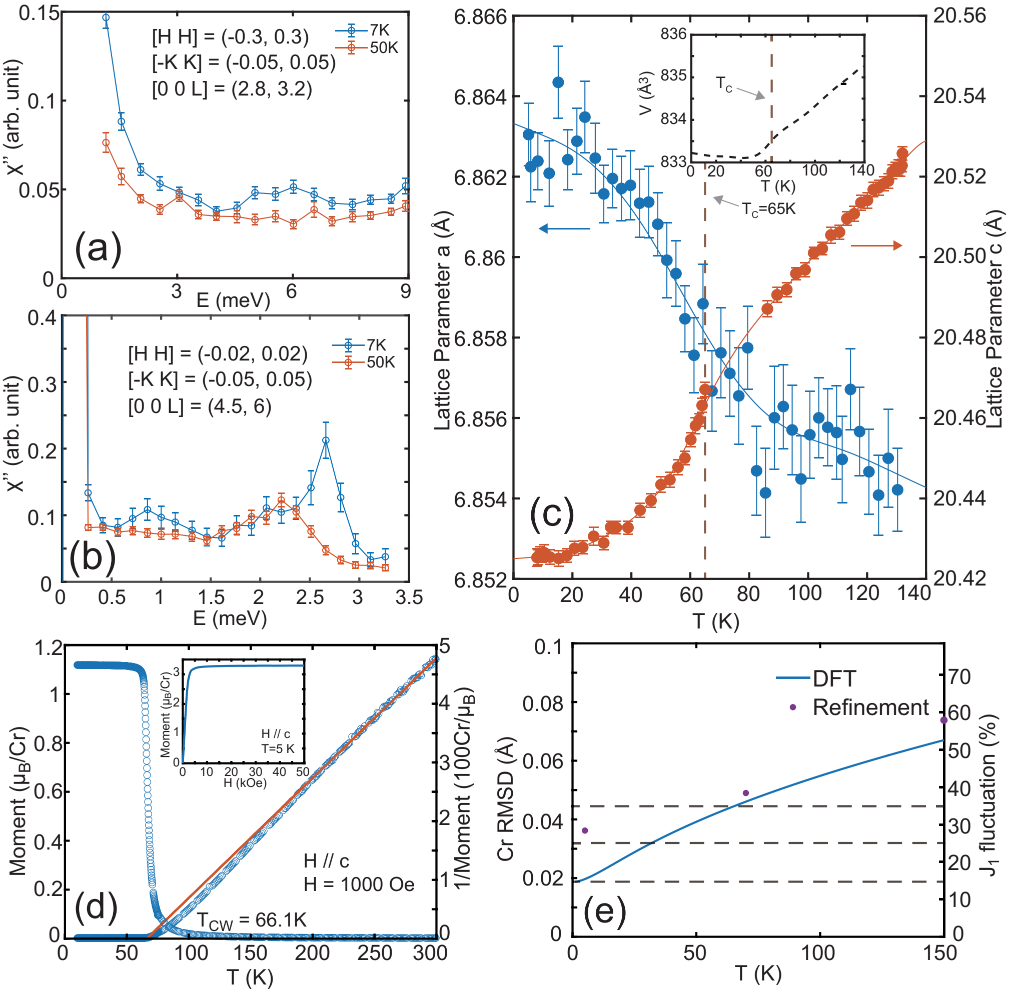

Figure S5: **Temperature dependent neutron data and bulk susceptibility.** (a,b) Local dynamic susceptibility, $\chi''(E)$, at $T = 7$ and 50 K, respectively. In (a) and (b), the integration was done over a wide range in the in-plane and out-of-plane wave vectors, respectively. The exact ranges of the integration are marked inside the plots. The data were measured using the AMATERAS spectrometer. (c) Temperature dependences of the lattice parameters a and c, respectively, measured using the CORELLI diffractometer. The solid lines through data are guides to the eye. The inset shows the calculated temperature-dependent change of the hexagonal unit cell volume using the guides to the eye. (d) M-T plot of $CrGeTe_3$ with out of plane fields, the inset shows M-H relationship at 5K. (e) The root mean square displacement (RMSD) of Cr atom as a function of temperature, and its corresponding fluctuation effect on J_1. The gray dashed lines indicate J_1 fluctuations of 15%, 25% and 35%.

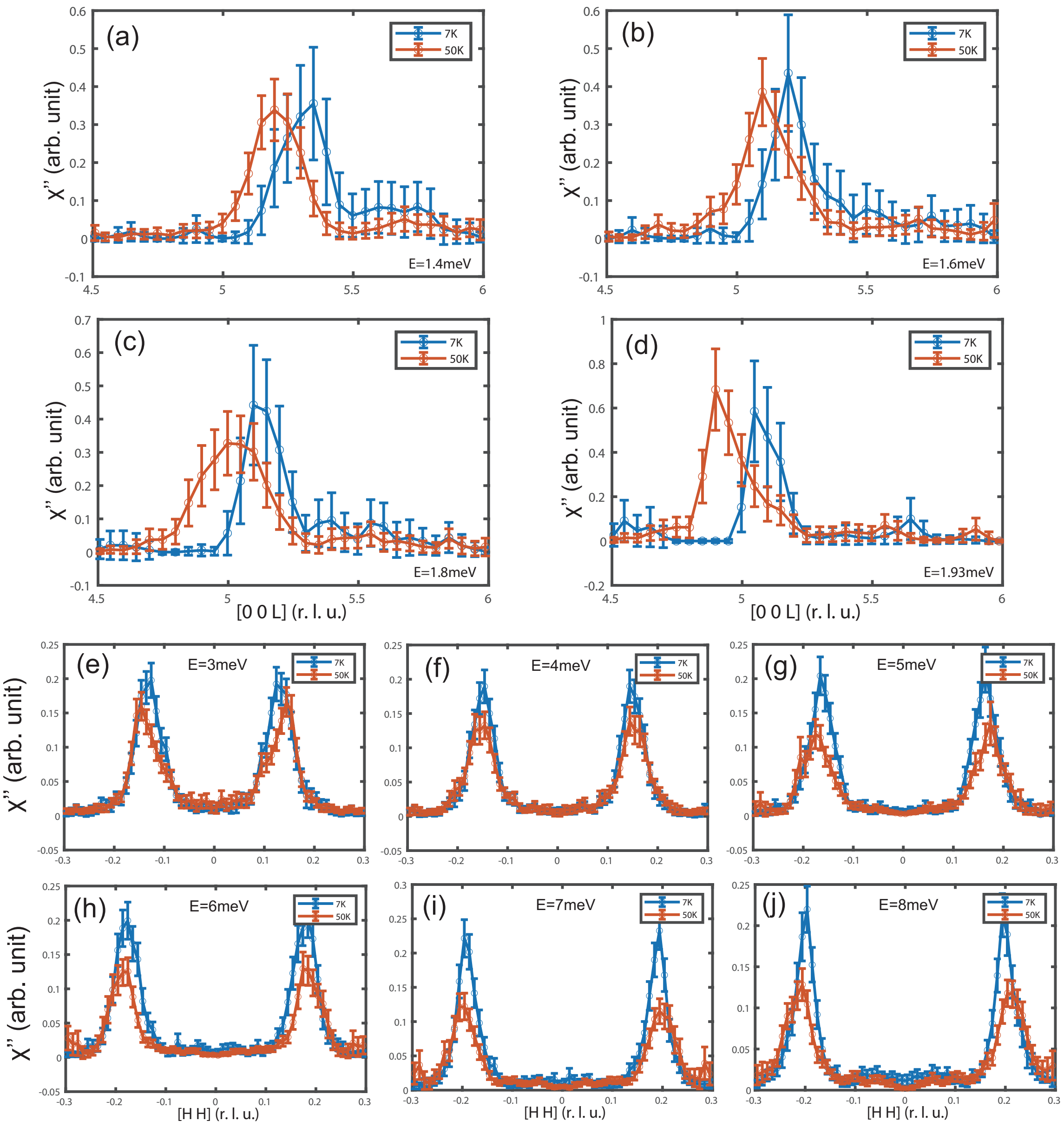

Figure S6: **Temperature dependent constant-E cuts at different wavevectors**. Const-E cuts showing the local dynamic susceptibility χ'' as a function of momentum transfer $\mathbf{Q}$ in (a-d) $[0,0,L]$ and (e-j) $[H,H]$ directions, respectively. The energy integration range is ± 0.1 meV and ± 0.5 meV for (a-d) and (e-j), respectively.

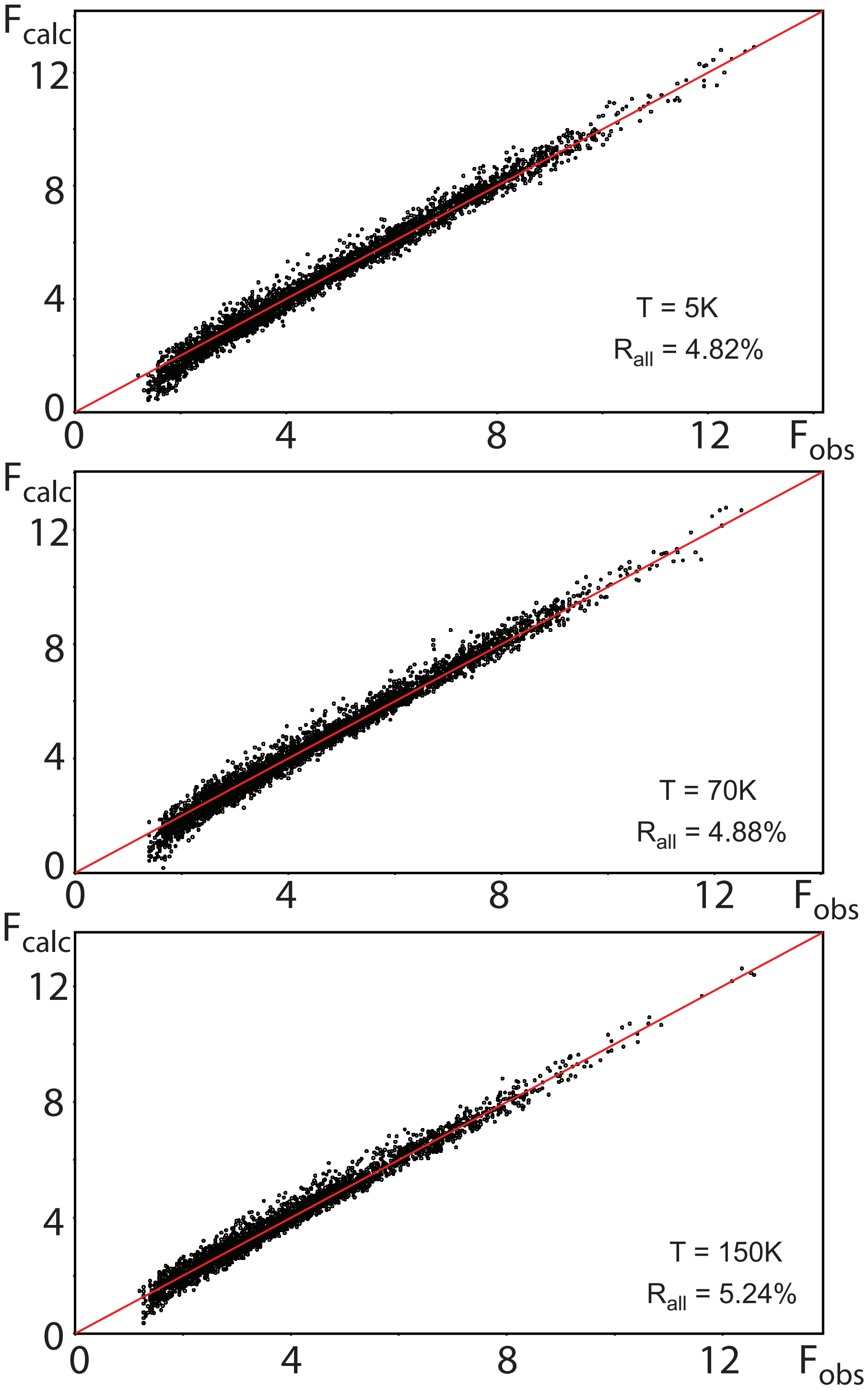

Figure S7: **Single crystal neutron refinement results.** Refinement results of the TOPAZ neutron diffraction experiment at (a) 5 K, (b) 70 K and (c) 150 K, respectively.

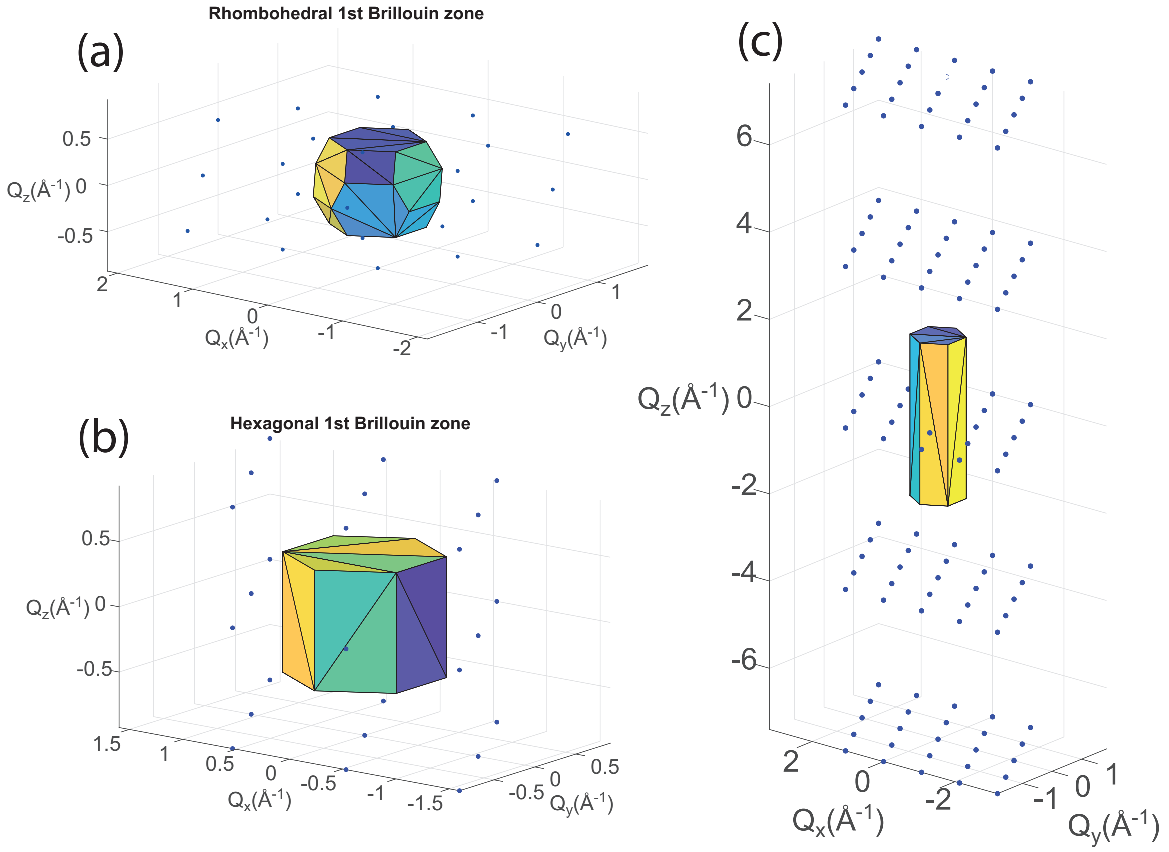

Figure S8: **Illustration of the integration range of the total moment sum rule calculations.** The left part shows the (a) rhombohedral and (b) hexagonal first Brillouin zone and panel (c) in the right shows the integration range for experiment and LSWT simulations. The axes are $Q_x \| [H\ 0]$, $Q_y \| [\text{-}K/2, K]$, and $Q_z \| [0\ 0\ L]$.
